# Diagnostic Accuracy of ChatGPT Model 5.1 in the Optical Characterization of Colorectal Lesions

**DOI:** 10.7759/cureus.111518

**Published:** 2026-06-25

**Authors:** Alejandro Osorio-Euan, Katia Lopez Garcia, Yoeli M Escandon Espinoza

**Affiliations:** 1 Gastrointestinal Endoscopy, Instituto de Seguridad y Servicios Sociales de los Trabajadores del Estado-Hospital Regional de Alta Especialidad Bicentenario de la Independencia, Tultitlan, MEX

**Keywords:** adenoma detection, artificial intelligence, chatgpt, colonoscopy, colorectal polyps, colorrectal cancer, gastrointestinal endoscopy, histopathology (hp)

## Abstract

Background

Optical characterization of colorectal lesions during colonoscopy is essential for determining the therapeutic strategy and predicting histology in real time. Artificial intelligence (AI) has emerged as a promising adjunctive tool to improve lesion characterization and reduce interobserver variability. Although computer-aided diagnosis systems have shown encouraging results, evidence regarding the performance of large language models in endoscopic lesion characterization remains limited.

Aim

The aim of this study is to evaluate the diagnostic accuracy of ChatGPT model 5.1 in differentiating adenomatous from non-adenomatous colorectal lesions during colonoscopy, using histopathology as the gold standard.

Methods

A retrospective diagnostic accuracy study was conducted including 93 colorectal lesions identified during colonoscopy. Optical descriptions of lesions were analyzed using ChatGPT 5.1, which classified lesions as adenomatous or non-adenomatous according to predefined criteria. Histopathological examination served as the reference standard. Diagnostic performance was evaluated through sensitivity, specificity, positive predictive value (PPV), negative predictive value (NPV), and overall accuracy using a 2×2 contingency matrix.

Results

Among 93 lesions, 35 (37.6%) were histologically confirmed adenomas. ChatGPT correctly identified 34 adenomas (true positives) and misclassified one lesion as non-adenomatous (false negative). Among non-adenomatous lesions, 46 were correctly classified (true negatives), while 12 were incorrectly categorized as adenomatous (false positives (FPs)). Sensitivity was 97.1%, specificity 79.3%, PPV 73.9%, NPV 97.9%, and overall diagnostic accuracy 86.0%. Most discrepancies occurred in lesions with inflammatory changes, superficial erosions, or mixed histologic patterns.

Conclusions

ChatGPT 5.1 demonstrated high sensitivity and excellent NPV for adenomatous lesion detection, suggesting potential utility as a supportive tool for optical diagnosis during colonoscopy. However, moderate specificity and the presence of FPs indicate that histopathological confirmation remains necessary before clinical implementation.

## Introduction

Colorectal cancer remains one of the leading neoplasms worldwide and a major cause of preventable mortality. Its development is largely associated with the progression of precursor lesions such as adenomas and serrated lesions, making colonoscopy a central tool for both early detection and prevention through endoscopic resection [1,2]. The quality of the procedure depends not only on cecal intubation or adequate bowel preparation, but also on the accurate identification of subtle lesions and the establishment of reliable optical characterization to guide therapeutic decision-making [3].

Optical characterization of colorectal polyps aims to predict the histologic nature of a lesion in real time based on its morphologic, vascular, and surface features. This approach is particularly useful for differentiating adenomatous from hyperplastic lesions, since adenomas possess premalignant potential and usually require resection, whereas some diminutive hyperplastic polyps, especially in the rectosigmoid colon, may be managed conservatively under strict criteria [4]. However, the diagnostic accuracy of optical characterization depends on the endoscopist’s expertise, image quality, the type of technology available, and the correct interpretation of classifications such as the Narrow-band Imaging International Colorectal Endoscopic Classification (NICE), Japan NBI Expert Team (JNET) Classification, or Workgroup on Serrated Polyps and Polyposis (WASP) Classification [5].

In this context, artificial intelligence (AI) has assumed an increasingly important role in gastrointestinal endoscopy. Computer-aided detection (CADe) systems were developed to identify possible polyps during real-time colonoscopy, whereas computer-aided diagnosis (CADx) systems aim to predict the histology of detected lesions [6]. Recent evidence suggests that CADe systems may increase adenoma detection rates in controlled studies; nevertheless, their impact in real-world settings appears more variable, and questions remain regarding their influence on hard clinical outcomes, such as reduction of interval colorectal cancer [7,8].

The European Society of Gastrointestinal Endoscopy has recognized the potential value of AI in endoscopy, particularly in the diagnosis and management of gastrointestinal neoplasia, while also emphasizing the need to validate these tools within frameworks of quality, safety, and clinical utility [9]. Similarly, recent guidelines have maintained a cautious position regarding the routine use of CADe systems, noting that although the technology may improve polyp detection, the magnitude of benefit, cost-effectiveness, and long-term clinical impact should be interpreted carefully [10].

Unlike commercial CADe and CADx systems, large language models such as ChatGPT were not originally designed as medical devices or dedicated endoscopic interpretation platforms. Nevertheless, their ability to process structured clinical information, integrate morphologic descriptions, and generate reasoned diagnostic responses has attracted growing interest across multiple areas of gastroenterology [11]. Recent studies have evaluated the performance of models such as ChatGPT in colonoscopy surveillance recommendations and clinical decision-making, showing promising results, although still insufficient to replace medical judgment or histopathologic standards [12].

The use of generative models in the characterization of colorectal lesions raises an important question: whether a structured endoscopic description can be interpreted by a language model with sufficient accuracy to differentiate adenomatous from non-adenomatous lesions. This possibility is especially attractive in settings where commercial CADx systems are unavailable because of economic or technological limitations. Nevertheless, diagnostic performance must be evaluated using objective metrics, including sensitivity, specificity, positive predictive value, negative predictive value, and overall accuracy, always using histopathology as the reference standard [13].

This study was previously presented as a scientific poster at Digestive Disease Week (DDW) 2026, held in Chicago, Illinois, USA, from May 2 to 5, 2026. An abstract of this study was published in the official DDW abstract supplement of Gastroenterology [14].

## Materials and methods

Study design and setting

A retrospective diagnostic accuracy study was conducted at the Endoscopy Unit of the Hospital Regional de Alta Especialidad del Bicentenario de la Independencia (HRAEBI-ISSSTE), Mexico. The study evaluated colorectal lesions identified during routine colonoscopic examinations.

Study population

A total of 93 colorectal lesions from patients undergoing colonoscopy were included. Lesions with available optical endoscopic characterization and histopathological confirmation were eligible for analysis. Lesions lacking definitive pathology or incomplete endoscopic documentation were excluded.

Optical characterization protocol

Each lesion underwent conventional optical evaluation during colonoscopy. Representative endoscopic images were collected, and morphologic and optical characteristics were documented using structured descriptions that included lesion size, location, morphology, vascular pattern, surface pattern, and overall endoscopic appearance.

The representative endoscopic images, together with their corresponding structured descriptions, were subsequently submitted to ChatGPT 5.1 (OpenAI, San Francisco, California, United States) for analysis. Based on the information provided, the model classified each lesion into one of two categories: (i) adenomatous lesion and (ii) non-adenomatous lesion (hyperplastic/non-polyp).

To facilitate statistical analysis and comparison with histopathologic findings, lesion classifications were converted into binary variables: adenoma = 1 and hyperplastic/non-adenomatous lesion = 0.

Histopathologic evaluation

Histopathologic diagnosis was established using specimens obtained from complete endoscopic resection of the lesions. Histopathology served as the reference standard for all analyses and was used to classify lesions as adenomatous or non-adenomatous.

Statistical analysis

Diagnostic accuracy analysis was performed using a 2×2 contingency matrix. The following diagnostic performance metrics were calculated: Sensitivity, specificity, positive predictive value (PPV), negative predictive value (NPV), and overall diagnostic accuracy.

True positives (TPs), true negatives (TNs), false positives (FPs), and false negatives (FNs) were determined by comparing AI predictions against histopathologic diagnosis. Descriptive statistics were expressed as absolute frequencies and percentages.

## Results

A total of 93 colorectal lesions were analyzed. Histopathologic examination confirmed 35 lesions (37.6%) as adenomas and 58 lesions (62.4%) as non-adenomatous.

ChatGPT 5.1 correctly identified 34 of 35 adenomatous lesions, yielding one FN classification. Among non-adenomatous lesions, the model correctly classified 46 lesions and generated 12 FP diagnoses.

The resulting diagnostic performance metrics are shown in Table 1. The model achieved a sensitivity of 97.1%, specificity of 79.3%, PPV of 73.9%, NPV of 97.9%, and an overall accuracy of 86.0% for differentiating adenomatous from non-adenomatous colorectal lesions.

**Table 1:** Diagnostic accuracy metrics of ChatGPT 5.1 in the characterization of colorectal lesions. Diagnostic performance metrics of ChatGPT 5.1 compared with histopathology. ChatGPT 5.1: OpenAI, San Francisco, California, United States

Metric	Value
Sensitivity	97.10%
Specificity	79.30%
Positive Predictive Value	73.90%
Negative Predictive Value	97.90%
Overall Accuracy	86.00%

Most diagnostic discrepancies occurred in lesions with inflammatory changes, superficial erosions, or mixed hyperplastic-adenomatous features.

**Figure 1:**
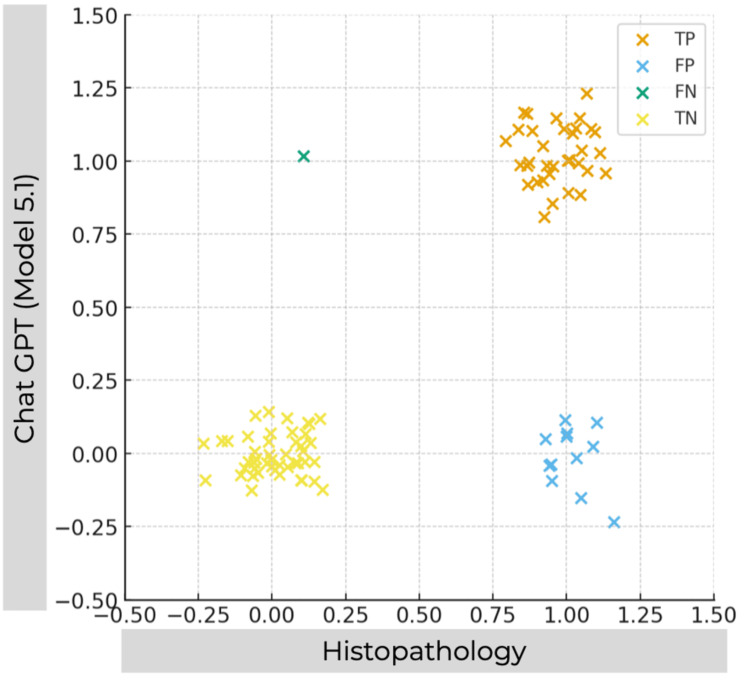
Scatter plot of ChatGPT 5.1 classifications compared with histopathology. Each point represents an individual colorectal lesion classified as true positive (TP), true negative (TN), false positive (FP), or false negative (FN). The clustering of TP and TN cases reflects the high diagnostic performance of ChatGPT 5.1, whereas the limited number of FN cases contributes to the model’s high sensitivity for adenoma detection. ChatGPT 5.1: OpenAI, San Francisco, California, United States

The dispersion analysis demonstrated clear clustering of TP and TN classifications, with limited overlap between groups. FP cases were predominantly located among lesions with atypical inflammatory or mixed-pattern characteristics (Figure 1).

## Discussion

This study demonstrated that ChatGPT 5.1 achieved high sensitivity and excellent NPV in the optical characterization of colorectal lesions. These findings suggest that generative AI models may have potential utility as supportive tools during colonoscopy, particularly for identifying adenomatous lesions with low probability of missed neoplasia.

The sensitivity observed in the present study (97.1%) is comparable to results previously reported for dedicated CADx systems using deep learning algorithms. Prior studies evaluating AI-assisted optical diagnosis have reported sensitivities ranging from 90% to 98% for adenomatous lesion detection, supporting the concept that artificial intelligence can contribute to real-time endoscopic decision-making [4,5].

The high NPV observed in this study is clinically relevant because it indicates a low probability of missed adenomas when the model classifies a lesion as non-adenomatous. This characteristic could theoretically support future “diagnose-and-leave” or “resect-and-discard” strategies if validated prospectively under standardized conditions [4,9].

However, specificity remained lower than sensitivity, primarily because of FP classifications involving inflammatory or mixed lesions. This finding suggests that ChatGPT may overinterpret atypical surface or vascular patterns as adenomatous features. Similar limitations have been reported in other AI-based optical diagnosis systems, particularly when lesions present inflammatory changes, erosions, or hybrid histologic patterns [4,5].

The relatively high FP rate may be partially attributable to variability in the interpretation of lesion characteristics and in the documentation of endoscopic findings. Although representative endoscopic images were provided to the model, diagnostic performance may also have been influenced by differences in the accompanying structured descriptions, including lesion size, morphology, vascular pattern, and surface pattern. Future studies should employ predefined classification systems such as NICE, JNET, and WASP to improve standardization, reproducibility, and interobserver consistency.

Unlike conventional CADx systems trained exclusively on large endoscopic image datasets, ChatGPT 5.1 was evaluated as a multimodal model that received both representative endoscopic images and structured lesion descriptions. Consequently, its performance may depend not only on image interpretation but also on the quality, structure, and completeness of the clinical information provided. This introduces potential variability that should be considered when interpreting the results and comparing them with dedicated image-based artificial intelligence platforms [11,12].

An important strength of this study is the use of histopathology as the reference standard. Additionally, the study explores a novel application of generative AI in gastrointestinal endoscopy, an area with limited available evidence. Nevertheless, several limitations should be acknowledged [11].

First, the retrospective design may introduce selection bias. Second, the sample size was relatively small and derived from a single center. Third, optical characterization was based on structured descriptions rather than direct image analysis. Finally, the study did not evaluate interobserver variability or compare ChatGPT performance directly against expert endoscopists.

Future prospective multicenter studies should evaluate the performance of generative AI models using standardized endoscopic imaging, larger datasets, and direct comparisons with established CADx platforms. Further research should also assess reproducibility, workflow integration, cost-effectiveness, and medico-legal implications before clinical adoption can be considered [6,7,9,10,13].

## Conclusions

ChatGPT 5.1 demonstrated high diagnostic sensitivity and excellent NPV for adenomatous colorectal lesion characterization. These findings support its potential role as an adjunctive tool for real-time optical diagnosis during colonoscopy. However, moderate specificity and FP classifications indicate that histopathological confirmation remains essential. Larger prospective studies are required to validate these findings and determine the clinical applicability of generative AI models in endoscopic practice.

## References

[1] Sung H, Ferlay J, Siegel RL, Laversanne M, Soerjomataram I, Jemal A, Bray F (2021). Global Cancer Statistics 2020: GLOBOCAN estimates of incidence and mortality worldwide for 36 cancers in 185 countries. CA Cancer J Clin.

[2] Shaukat A, Kahi CJ, Burke CA, Rabeneck L, Sauer BG, Rex DK (2021). ACG clinical guidelines: colorectal cancer screening 2021. Am J Gastroenterol.

[3] Rex DK, Boland CR, Dominitz JA (2017). Colorectal cancer screening: recommendations for physicians and patients from the U.S. Multi-Society Task Force on colorectal cancer. Gastroenterology.

[4] Mori Y, Hassan C (2025). Computer-aided diagnosis of colorectal polyps: assisted or autonomous?. Clin Endosc.

[5] Cheng Y, Li L, Bi Y (2024). Computer-aided diagnosis system for optical diagnosis of colorectal polyps under white light imaging. Dig Liver Dis.

[6] Hassan C, Spadaccini M, Mori Y (2023). Real-time computer-aided detection of colorectal neoplasia during colonoscopy: a systematic review and meta-analysis. Ann Intern Med.

[7] Patel HK, Mori Y, Hassan C (2024). Lack of effectiveness of computer aided detection for colorectal neoplasia: a systematic review and meta-analysis of nonrandomized studies. Clin Gastroenterol Hepatol.

[8] Sultan S, Shung DL, Kolb JM (2025). AGA living clinical practice guideline on computer-aided detection-assisted colonoscopy. Gastroenterology.

[9] Messmann H, Bisschops R, Antonelli G (2022). Expected value of artificial intelligence in gastrointestinal endoscopy: European Society of Gastrointestinal Endoscopy (ESGE) Position Statement. Endoscopy.

[10] Bretthauer M, Ahmed J, Antonelli G (2025). Use of computer-assisted detection (CADe) colonoscopy in colorectal cancer screening and surveillance: European Society of Gastrointestinal Endoscopy (ESGE) Position Statement. Endoscopy.

[11] Rey JF (2024). As how artificial intelligence is revolutionizing endoscopy. Clin Endosc.

[12] Chang PW, Amini MM, Davis RO (2024). ChatGPT4 outperforms endoscopists for determination of postcolonoscopy rescreening and surveillance recommendations. Clin Gastroenterol Hepatol.

[13] Shinozaki S, Watanabe J, Kanno T, Yuan Y, Yano T, Yamamoto H (2025). Computer-aided diagnosis for colorectal polyp in comparison with endoscopists: systematic review and meta-analysis. Dig Endosc.

[14] Osorio-Euan A, Espinoza Y, García K, Perez-Roa GF, Avila-Franco AY, Balderas-Ortega JC (2026). Diagnostic accuracy of ChatGPT model 5.1 in the characterization of colorectal lesions. Gastroenterology.

